# The role of digital financial inclusion in China on urban—rural disparities in healthcare expenditures

**DOI:** 10.3389/fpubh.2024.1397560

**Published:** 2024-08-02

**Authors:** Yuyang Zhang, Keyi Li, Yumeng Pang, Peter C. Coyte

**Affiliations:** ^1^School of Economics, Qingdao University, Qingdao, China; ^2^Melbourne Graduate School of Education, The University of Melbourne, Melbourne, VIC, Australia; ^3^School of Public Finance and Taxation, Nanjing University of Finance and Economics, Nanjing, China; ^4^Institute of Health Policy, Management and Evaluation, University of Toronto, Toronto, ON, Canada

**Keywords:** digital financial inclusion, healthcare, urban–rural disparities, government health expenditures, moderating effect

## Abstract

**Introduction:**

The digital financial inclusion (DFI) provides opportunities to improve the relative capacity to pay for healthcare services by rural residents who are usually underserved by traditional finance in China. This paper provides empirical evidence on how the development of DFI affects the healthcare expenditure disparities between urban and rural residents.

**Methods:**

We employed the fixed effects model and instrumental variable method to estimate the impact of DFI on the Theil index of urban–rural disparities in healthcare expenditures, using panel data from 31 provinces (2011 ~ 2020) in China. We further adopted a moderating effect model to test whether the intensity of the impact would vary depending on the level of local government health expenditures.

**Results:**

The results suggest a negative association between the development level of DFI and the urban–rural healthcare expenditure disparities in China. For every 1% increase in the DFI index, the Theil index of urban–rural disparities in healthcare expenditures would fall by 0.0013. After changing the measurement method for the dependent variable and adjusting the sample, the results remain robust. Moreover, the result of the moderating effect model indicates that, a high level of government health expenditures is conducive to the impact of DFI.

**Discussion:**

Our research reveals that DFI plays an important role in bridging the urban–rural gap in healthcare expenditures. This finding provides new information for addressing the issue of urban–rural healthcare inequality in China. Chinese government needs to accelerate the construction of digital infrastructure and increase the penetration rate of digital tools in rural areas to promote the beneficial effects of DFI. Additionally, it is also necessary for local government to address the unbalanced allocation of medical resources between urban and rural areas, especially the shortage of rural human resources.

## 1 Introduction

Access to quality healthcare is important for both health and human capital investments, and therefore, is a crucial factor to foster social equity by narrowing the gap in healthcare service use between urban and rural residents in China. As mentioned in the Outline of “Healthy China 2030” Plan issued by the Chinese government in 2016, the construction of a healthy China should focus on the promotion of urban–rural equalization of the use of healthcare services. However, Data from the China Statistical Yearbook indicates that per capita healthcare expenditures by urban residents was more than 59% higher than that for rural residents in 2021. Previous literature has shown that people’s health is positive correlated with their socioeconomic status (1, 2). Due to China’s urban–rural dual system, the socioeconomic status of rural residents is generally inferior to that of urban residents (3). This situation means that rural residents have worse health status, thus larger potential demand for healthcare. Therefore, addressing the contradiction between large potential demand and low utilization of healthcare among rural residents is a big challenge in promoting urban–rural integration in China.

Credit constraints and high healthcare costs are considered important reasons for the lack of healthcare among the poor in low- and middle-income countries (4). The digital financial inclusion (DFI) provides opportunities to improve the relative capacity to pay for healthcare services by rural residents who are usually underserved by traditional finance. DFI represents a combination of traditional forms of inclusive finance and modern forms of digital technology (5). The concept of Inclusive Finance was first proposed by the United Nations in 2005, and refers to the provision of sustainable financial services to all social classes and groups with equal opportunities and affordable costs. Its key service targets are vulnerable groups unable to access or lack financial services, such as small and micro enterprises, rural residents, and low-income groups. Initially, inclusive finance took the form of non-profit microcredit, and later gradually expanded to comprehensive finance that included multiple forms of payment and credit services. Furthermore, with the increasing development of digital technology (such as information technology, Big Data and Cloud Computing), inclusive finance has developed into the so-called DFI. The application of digital technology has greatly eased the cost constraints of financial services, endowing DFI with more obvious characteristics of low cost, high coverage, and sustainability, which greatly contrasts with traditional forms of finance. Traditional finance needs to increase its coverage by setting up institutional branches, ATMs and other hardware facilities. Moreover, to achieve self-development and profitability, these financial resources are more likely to be allocated in densely populated and affluent areas, and to the rich. Therefore, traditional finance has obvious pro-rich consequences (6). In contrast, DFI allows customers to access financial services through various devices such as computers and mobile phones, often without the need to interact with traditional financial facilities. It has also lowered customer entry barriers through product innovation, making the trend of financial services more affordable and overcoming the pro-rich disadvantage of traditional finance. In 2016, the G20 High-Level Principles for Digital Financial Inclusion was submitted by China, elevating the development of digital inclusive finance to a national strategic level.

Existing literature has found that DFI can enhance the economic welfare of financially underserved populations. It is mainly because that access to financial services could help the poor obtain capital accumulation (7), and provide them more opportunities for transactions and savings (8). Greater attention has recently been paid to the effect of DFI on the promotion of the coordinated development of economic welfare in urban and rural areas. Previous literature on the impact of DFI on China’s urban–rural disparities generally shows that DFI can effectively reduce the urban–rural income gap (9, 10). There are also a series of studies exploring the impact of DFI on consumption inequality (11) as consumption expenditures may offer a better indicator of welfare compared to income (12), especially for resource-poor families in rural households (13). In contrast to research on the effects of DFI on income, there is still a lack of consensus of the impact of DFI on the urban–rural consumption gap. Some studies suggest DFI might narrow this gap (14–16) as it helps rural residents use financial services. DFI not only enhances the economic capability of individuals, it also alleviates their liquidity constraints, thus smoothing consumption across different periods and stimulating current consumption demand (17). For urban residents who have already benefited from the traditional finance, the marginal influence of DFI on them may not be as significant as that for rural residents. In contrast, He and Song (18) found that due to the urban–rural differences of supply-side goods market, DFI mainly promotes urban consumption, so it has no significant effect on urban–rural gap.

Most of the existing literature focuses on urban–rural disparities in overall consumption rather than on a specific category of consumption expenditures. However, DFI may have heterogeneous effects on various types of expenditures (14, 19). In the few related studies, Zhang and Cai (20) found that DFI has no significant impact on the urban–rural gap in survival expenditures such as food, but significantly reduces the gap in development expenditures such as education and healthcare; while Li and Zheng (21) got the opposite conclusion that DFI widened the urban–rural gap in entertainment and healthcare expenditures. Additionally, healthcare expenditures are potentially different from other categories of consumption expenditures, as healthcare expenditures are usually related to individuals’ illnesses and health conditions. The classic theory of health demand proposed by Grossman (22) views health as a durable capital stock, assuming that the inherited initial stock of health depreciates with age and can be increased by investment. In this model, healthcare is the most important investment goods and people purchase this scarce resource within their limited budget constraints. So individuals acquire healthcare services to the point at which the potential gains in health status generate sufficient improvements in lifetime well-being relative to the cost of those healthcare services. The theory suggests that the demand for healthcare services is a derived demand as it depends exclusively on how productive such services are in advancing health status. Moreover, since individuals’ access to healthcare services is constrained by their budget, policies that help loosen liquidity constraints, such as DFI, may release their healthcare needs and increase healthcare expenditures, which is the initial motivation behind our research topic. This paper aims to answer the following questions: Can China’s DFI alleviate the urban–rural disparities in healthcare expenditures? What is the potential mechanism? The existing literature indicates that these answers are not yet clear.

## 2 Theoretical analysis

### 2.1 DFI and urban–rural disparities in healthcare expenditures

According to Grossman’s theory (22), as income represents a limit on resources to employ to advance health status, the model predicts that individuals with higher incomes would both spend more on healthcare services and command a higher health status. The positive relationship between income and healthcare expenditures has been consensus in many studies (23, 24). DFI enables rural residents who were previously excluded from traditional finance to use financial resources for production and investment, so as to obtain more income, while urban residents who are relatively wealthy have already benefited from the traditional finance, so the development of DFI may have a more obvious marginal effect on rural residents. After the popularization of DFI, the income level of rural residents may rise more relatively, thus narrowing the urban–rural gap in healthcare expenditures.

In addition, if DFI were to increase access to various financial services that increase the resources available for individuals to allocate on healthcare services, then this would be akin to an increase in income and would be associated with increased healthcare expenditures. Given their exposure to traditional forms of finance, urban residents will gain less through DFI than their rural counterparts. This suggests that DFI will tends to narrow disparities in healthcare expenditures between rural and urban residents. Specifically, DFI can provide rural residents with digital payment, credit, transfer and insurance services, and so on. First, the development of DFI makes it universal to use mobile phones and other devices to conduct digital payment by scanning code. Compared with traditional forms, the convenience of payment is greatly improved. Second, DFI can help rural residents access formal and informal credit. A study based on Chinese households showed that digital finance could promote healthcare utilization, as the improved accessibility of online credit effectively relieves consumers from the constraints of micro loans (14). Consumer credit products on formal digital financial platforms such as Alipay’s Huabei make it easier to obtain loan services. This is not only because digital financial platforms can overcome geographical constraints (25), but also because technologies such as big data and cloud computing allow these platforms to know the real repayment ability and willingness of the users, thus making up for the lack of credit evaluation of rural residents caused by information asymmetry. Relevant studies have confirmed that China’s DFI can effectively alleviate the credit constraints of rural residents (26, 27). Meanwhile, digital transfer service may help rural residents subject to liquidity constraints to obtain informal credit in time. An empirical research based on South Africa showed that the mobile money transfer technology can promote people to use necessary healthcare through greater ease of informal borrowing (28). In a society like China that values kinship relationships, a series of studies have explored this viewpoint, which found that digital transfer service can promote healthcare expenditures by increasing social interactions and borrowing among family and friends (29, 30). Third, DFI also includes digital insurance service. According to data from the National Medical Insurance Administration, by the end of 2023, the number of digital medical insurance users in China had exceeded 1 billion, with all 31 provinces supporting the use of digital medical insurance for medical treatment and drug purchase, and more than 800,000 designated medical institutions connected. The popularity of digital medical insurance makes it more convenient for rural residents to participate in insurance, pay fees and reimburse, which may also promote their use of healthcare services.

### 2.2 The impact of government health expenditures

It can be seen from the study of He and Song (18) that, whether DFI can influence the urban–rural disparities in healthcare expenditures depends not only on the urban–rural capacity gap to pay on the demand-side, but also on the urban–rural gap in medical resource allocation on the supply-side, which is related to the level of government health expenditures. It means that government’ public financial support on healthcare system may play an important role in the effect of DFI. According to the Andersen (31) model, enabling factors are key factors that affect healthcare use, including the economic capacity of individuals or households and supply-side factors of medical resources. Therefore, residents’ healthcare expenditures are not only affected by the purchasing power of the demand side, but also related to supply-side factors such as the completeness of the healthcare service system and the accessibility of medical resources.

There are three main directions of China’s government health expenditures: the basic medical security system, the medical service supply system, and the public health system. According to the data from China Health Statistical Yearbook, more than 85% of China’s government health expenditures flowed to the former two directions from 2011 to 2020. On one hand, China’s basic medical security system has significantly reduced the relative price of healthcare. Therefore, a more comprehensive medical security system may be more conducive to the promotion of DFI on healthcare expenditures. For rural residents who lack medical security, the marginal effect of this effect may be stronger than that of urban residents with relative advantages. On the other hand, the government’s financial support in the medical service supply system not only increases the number of medical institutions, but also greatly improves the quality of medical services. Its direct effect is to enhance residents’ accessibility to high-quality medical resources, thereby crowding in healthcare expenditures. Especially since the new medical reform in 2009, Chinese government has intensified its financial support of medical service supply system in rural areas. Therefore, government health expenditures may also improve the accessibility of rural medical resources, thereby further strengthening the impact of DFI. Based on the analysis of the above two aspects, we propose the following hypothesis: the effect of DFI on the urban–rural gap in healthcare expenditures may be moderated by government health expenditures, i.e., the higher the level of government health expenditures, the stronger the alleviating effect of digital financial inclusion on the urban–rural gap in healthcare expenditures. We will use a moderating effect model in the empirical research section to verify this conjecture.

## 3 Materials and methods

### 3.1 Data

Data were derived from two sources: the Peking University Digital Financial Inclusion Index of China (PKU-DFIIC) (2011 ~ 2020) compiled by a research team who are from the Institute of Digital Finance at Peking University, Guo et al. (32), and province-level panel data (2011 ~ 2020) from the China Statistical Yearbook. The PKU-DFIIC accurately characterizes the development of DFI in different regions of China, using a large micro-dataset provided by Ant Financial Services Group, a dominant firm in China’s digital finance industry. This database can be publicly downloaded from the website of Peking University Digital Research Center^1^. Our sample included 31 provinces, municipalities and autonomous regions, excluding Taiwan Province, Hong Kong and Macao Special Administrative Region. The analyses were performed on a balanced set of data with 310 observations.

### 3.2 Variables

#### 3.2.1 Dependent variable

Urban–rural disparities in healthcare expenditures was the dependent variable. In general, the Gini Index and Theil Index are both commonly used indicators to measure inequality. However, the Gini Index is usually suitable for measuring overall inequality, while the Theil Index can measure both overall inequality and intergroup inequality due to its better decomposability. So following previous studies (33), we adopted the Theil Index to measure the degree of disparities between urban and rural residents’ healthcare expenditures. The Theil Index, also known as Theil’s Entropy Measure, was initially proposed by Theil (34) using the concept of entropy from information theory to calculate income inequality. Theil index ranges from 0 to 1, with higher values indicative of a more unequal distribution. It is defined as follows:


(1) $$Theil=\overset{2}{\underset{j=1}{\sum }}\left(\frac{E_{ijt}}{E_{it}}\right)\ln\left[\left(\frac{E_{ijt}}{E_{it}}\right)/\left(\frac{P_{ijt}}{P_{it}}\right)\right]$$

Urban and rural residents are designated by *j* where *j* = 1 signifies urban residents and *j* = 2 signifies rural residents. The subscript *i* and *t* represent province and year, respectively. The term, *E_ijt_*, denotes total healthcare expenditures in province, *i*, at time, *t*, for residents in group *j*. The term, *E_it_*, denotes total healthcare expenditures for both urban and rural residents in province, *i*, at time, *t*. The resident population *j* in province, *i*, at time, *t*, is denoted by *P_ijt_*, while *P_it_* represents the total population of province *i* at time *t*. Healthcare expenditures (yuan) and population data were obtained from the China Statistical Yearbook. Healthcare expenditures refer to “the total cost of medicines, medical products and services for healthcare” incurred by residents. Healthcare expenditures were inflation adjusted using the Consumer Price Index (CPI) of each province and presented in 2011 yuan.

Table 1 reports the comparison of per capita healthcare expenditures between urban and rural areas, and the mean values of urban-rural Theil index of 31 provinces in China according to Equation (1). The statistical results show that per capita healthcare expenditures for urban residents is significantly greater than that for rural residents. In terms of temporal trends, there exists an upward trend in per capita healthcare expenditures for all residents. It indicates that urban and rural residents’ real utilization level of healthcare has gradually improved. The urban–rural Theil index shows a decreasing trend, except for 2016 when there was a slight rebound. Overall, the index fell from 0.078 in 2011 to 0.026 in 2020 highlighting a decline in disparities. It should be noted that healthcare expenditures’ proportion in total expenditures for rural residents is always higher than that for urban residents. It to some extent indicates that rural residents have a higher relative demand for healthcare. The increasing share of healthcare expenditures of residents means the increase of healthcare demand, and there is a greater increase for rural residents. To sum up, although the urban–rural Theil index has been a decreased, there is still a large gap between urban and rural residents in terms of the absolute level of healthcare expenditures.

**Table 1:** Healthcare expenditures (HCE) of urban and rural residents in China 2011 ~ 2020.

Year	Urban residents		Rural residents		Urban–rural Theil index (mean)
	HCE per capita	Proportion in total expenditures	HCE per capita	Proportion in total expenditures	
2011	968.98	6.39%	436.75	8.37%	0.078
2012	1063.68	6.38%	513.81	8.70%	0.066
2013	1118.26	6.05%	614.20	8.21%	0.055
2014	1305.60	6.54%	753.90	8.99%	0.041
2015	1443.40	6.75%	846.00	9.17%	0.036
2016	1630.80	7.07%	929.20	9.17%	0.039
2017	1777.40	7.27%	1058.70	9.66%	0.038
2018	2045.70	7.83%	1240.10	10.23%	0.033
2019	2282.70	8.13%	1420.80	10.66%	0.029
2020	2172.20	8.04%	1417.50	10.34%	0.026

#### 3.2.2 Independent variable

The key independent variable used in this paper is the DFI index, which is extracted from the Peking University Digital Financial Inclusion Index of China (PKU-DFIIC) (2011 ~ 2020) compiled by Guo et al. (32), as mentioned in the data section. This index has been used extensively in a series of related empirical studies to date (11, 14). According to the PKU-DFIIC, the DFI index consists of three sub-indices, i.e., breadth of coverage, depth of usage, and degree of digitization, with a total of 33 specific indicators. The breath of coverage measures the population covered by digital financial services, which means that the greater the breadth of coverage, the more people it covers. It is measured by account coverage, including three indicators, i.e., the number of Alipay accounts per 10,000 people, the proportion of Alipay users who have linked their bank cards, and the average number of bank cards linked to per Alipay account. The depth of usage comprehensively calculates the actual number of users and usage intensity of six services (payment, insurance, monetary funds, investment, credit investigation and credit), including a total of 20 indicators. The greater depth of usage means that the users are more active and the financial services they use are more diversified. The degree of digitization uses 10 indicators to calculate the mobility, affordability, creditization and facilitation level of digital finance, which are measured by the proportion of mobile payment transactions and transaction amount, the average loan interest rate for small and micro business owners and individuals, the proportion of credit deposit free transactions and transaction amount, and the proportion of QR code payment transactions and transaction amount, respectively. The degree of digitization reflects the digital features of DFI. The higher the digitization degree, the more obvious the advantages of low cost and low threshold of digital financial services.

The DFI index and its sub-indices have three regional levels: province, city and county. We used the provincial-level indices in the regression analyses. To identify the main sources of DFI’s influence, we further examined the impact of three primary sub-indices as independent variables in the empirical study.

#### 3.2.3 Control variables

In order to obtain reliable estimation results, we added as many control variables as possible to minimize endogeneity issues caused by missing variables. According to the accessible data, the control variables designed in this paper are as follows: First, economic factors. The local economic development not only affects the level of residents’ consumption, but also has a positive correlation with the degree of digital reform. We controlled the real GDP per capita of each province converted to 2011, which was calculated using the annual GDP deflator. In addition to the overall economic development level, we also controlled for the differences in economic status between urban and rural residents, which is measured by urban–rural ratio of per capita disposable income. Second, government health expenditures. The China Statistical Yearbook provides annual data of per capita government budget expenditure on health. We used CPI to calculate the real value of per capita government budget expenditure. Third, urban–rural disparities in medical resources. As for the data of medical resources, the China Statistical Yearbook provides the number of health professionals and beds in medical institutions per thousand population in each province. After conducting a correlation test between urban–rural ratio of healthcare professionals and that of beds, it was found that the correlation coefficient was 0.8668 (greater than the critical value of 0.8). It indicates an obvious collinearity between the two variables. Therefore, we choose one of the two variables, i.e., urban–rural ratio of healthcare professionals, as the proxy variable for urban–rural disparities in medical resources. Fourth, social medical insurance participation. The level of healthcare expenditures is also affected by the price of healthcare products and services. Social medical insurance can effectively reduce the relative price of healthcare and improve the capacity to pay of residents, thus influence healthcare expenditures. China’s medical insurance systems include two types of insurances, one is the Employee Basic Medical Insurance scheme designed for employed residents, the other is the Resident Basic Medical Insurance scheme designed for non-employed residents. Considering the different coverage groups and benefit levels of various medical insurance systems, we, respectively, employed the participation rate of both types of medical insurance in each province as the proxy variables. Fifth, demographic factors. According to Grossman (22) model, the rate at which health capital depreciations is considered to grow with age. So the demand for healthcare services is also likely to be related to the population’s age structure. We used the older adult dependency ratio as the proxy variable, which is the ratio of older adult population (65 years old and above) to working-age population (15 ~ 64 years old). In addition, existing studies suggest that urbanization rate is a key variable affecting the urban–rural consumption gap in China (35). It is mainly because that, the migration of the rural labor force to urban areas may lead to changes in the economic structure, and the concentration of population in urban areas can also affect the urban–rural resource allocation. We used the ratio of urban population to total population to measure the variable of urbanization rate. Finally, given that environmental pollution may be related to the incidence of many diseases and consequently affect healthcare expenditures, we also controlled sulfur dioxide emissions per square kilometer (t/km^3^) as a proxy for the levels of pollution.

The descriptive statistics of all the variables are shown in Table 2.

**Table 2:** Variable descriptive statistics.

Variables	Obs.	Mean	SD	Min	Max
Theil index	310	0.04	0.06	7.04 × 10^−6^	0.54
DFI index	310	216.24	97.03	16.22	431.93
Breadth of coverage	310	196.67	96.56	1.96	397.00
Depth of usage	310	211.12	98.19	6.76	488.68
Degree of digitization	310	290.14	117.25	7.58	462.23
Per capita GDP	310	39380.94	18943.59	16,413	103610.9
Urban–rural ratio of per capita income	310	2.64	0.42	1.85	3.98
Per capita government health expenditures	310	901.47	397.76	372.85	3170.04
Urban–rural ratio of healthcare professionals	300	2.49	0.71	1.06	5.40
Participation rate of EBMI (%)	310	22.38	13.81	8.22	79.56
Participation rate of RBMI (%)	310	40.42	29.88	6.18	96.40
Older adult dependency ratio	310	0.14	0.04	0.07	0.25
Urbanization rate	310	0.58	0.13	0.23	0.90
Sulfur dioxide (SO_2_) emissions	310	3.47	4.95	0.003	37.87

### 3.3 Empirical models

We first estimated the impact of DFI index on urban–rural Theil index in healthcare expenditures. The baseline model is as follows:


(2) $$Theil_{it}=\alpha +\beta \ln DFI_{it}+\gamma Control_{it}+\lambda_{t}+\mu_{i}+\varepsilon_{it}$$

Where *Theil_it_*, ln*DFI_it_*, and *Control_it_* denote the province *i*’ urban–rural Theil index of healthcare expenditures, the logarithmic form of DFI index and a series of control variables, respectively; Coefficient *β* represents the average effect of logarithmic DFI index on the disparities between urban and rural healthcare expenditures; *α*, *γ*, *λ_t_*, *μ_i_*, and *ε_it_* represent the constant term, the estimated coefficients of the control variables, the time-fixed effect, the province-fixed effect, and the error term, respectively.

To alleviate possible endogenous problems caused by omitting variables, measurement errors and reverse causality, we have made the following efforts. First, we control as many factors as possible that may affect the dependent variable to alleviate endogeneity stemming from omitting variables. Second, the two-way fixed effects model and clustering robust standard errors are adopted to avoid endogeneity caused by unobserved fixed factors and measurement error. The third one is to employ an instrumental variable to identify potential causality using 2SLS method, which is still necessary to further overcome the endogenous bias. Referring to Luo and Li (11), this paper constructed a Bartik (36) instrumental variable as ln*DFI*_*i,t*-1_ × Δln*DFI_t,t-1_*, representing the product of the lagged logarithmic DFI index and the difference of the national overall logarithmic DFI index between the year of *t* and *t*−1. This instrumental variable is valid since it satisfies both correlation and exogeneity conditions. For the correlation condition, the lagged logarithmic DFI index of province *i* and the national overall logarithmic DFI index are both correlated with the logarithmic DFI index of province *i*. For the exogeneity condition, the Bartik instrumental variable, i.e., ln*DFI_i,t-1_* × Δln*DFI_t,t-1_*, does not have an intuitively direct impact on the dependent variable through other channels, except for the only way of endogenous variables.

To further explore the mechanism, we constructed a moderating model as follows to estimate whether the effect of DFI is related to government health expenditures:


(3) $$\begin{array}{c} Theil_{it}=\gamma_{0}+\gamma_{1}\ln DFI_{it}+\eta \ln DFI_{it}\times \ln Hea_{it}+\gamma_{2}Control_{it}\\ +\lambda_{t}+\mu_{i}+\varepsilon_{it} \end{array}$$

The interaction term was constructed using the logarithmic DFI index and government health expenditures. The coefficient *η* represents the moderating effect of government health expenditures. If the *η* is significantly negative, it means that the government health expenditures have a significant moderating effect, and the reducing effect of DFI on the urban–rural disparities in healthcare expenditures will be strengthened by the increase of government health expenditures. If *η* is significantly positive, it means that the reducing effect of DFI will be weakened by the increase of government health expenditures. If *η* is not significant, it means that the effect of DFI is not related to government health expenditures.

## 4 Results

### 4.1 Impact of DFI on urban–rural disparities in healthcare expenditures

#### 4.1.1 Baseline results

Before regression estimation based on Equation (2), we first conducted Hausman test. The result shows that *p* < 0.001, which strongly rejects the random effects model, indicating that it is feasible to choose the fixed effects model for baseline estimates. The estimated result of fixed effects model is shown in column (1) of Table 3. It shows that the DFI index is negatively associated with the urban–rural disparities in healthcare expenditures at the significance level of 0.05. The estimated coefficient is −0.057, which means for every 1% increase in the DFI index, the urban–rural Theil index will decrease by 0.0006.

**Table 3:** Baseline estimates of the impact of DFI on urban–rural healthcare disparities.

	Fixed effects	Fixed effects + IV	
		First stage	Second stage
	(1) Theil index	(2) lnDFI	(3) Theil index
lnDFI	−0.057**		−0.126***
	(0.023)		(0.048)
Bartik instrumental variable		0.225***	
		(0.014)	
ln (per capita GDP)	0.018	0.067***	0.007
	(0.025)	(0.024)	(0.022)
ln (government health expenditures)	−0.086**	0.062**	−0.078**
	(0.042)	(0.029)	(0.034)
Urban–rural ratio of healthcare professionals	0.014	−0.002	0.013*
	(0.012)	(0.005)	(0.008)
ln (participation rate of EBMI)	−0.162*	0.011	−0.161***
	(0.082)	(0.041)	(0.056)
ln (participation rate of RBMI)	−0.003	0.007**	−0.002
	(0.004)	(0.004)	(0.003)
Older adult dependency ratio	0.176	−0.191	0.131
	(0.191)	(0.152)	(0.1303)
Urbanization rate	0.394**	0.807***	0.439**
	(0.166)	(0.193)	(0.179)
Urban–rural ratio of per capita income	0.005	0.046**	0.003
	(0.027)	(0.022)	(0.017)
Sulfur dioxide (SO_2_) emissions	−0.001**	0.001	−0.001**
	(0.000)	(0.001)	(0.000)
FE of Province	Yes	Yes	Yes
FE of year	Yes	Yes	Yes
Wald-*F* statistics		334.085	
Obs.	300	269	269
*R* ^2^	0.493		0.419

The observations were slightly reduced, due to missing values in the variable of healthcare professionals. Clustering robust standard errors in brackets; *p < 0.1, **p < 0.05, ***p < 0.01. The critical values of Wald-*F* are 16.38 (10%), 8.96 (15%), 6.66 (20%), 5.53 (25%).

Since the PKU-DFIIC did not provide the provincial-level DFI index in 2010, the observations of 2011 were excluded from the model when the lagged logarithmic DFI index was adopted, resulting in a decrease in the total sample size (from 300 to 269). The results based on 2SLS method are shown in column (2) ~ (3) of Table 3. The estimated coefficient of Bartik instrumental variable at the first stage is 0.225 significantly at the level of 0.01. It represents that the instrumental variable is significantly correlated to the independent variable, satisfying the necessary validity condition of instrumental variable. The Wald-*F* statistics is 334.085, indicating that there is no weak identification problem, which means that this instrumental variable is reasonable statistically. At the second stage, the estimated coefficient of lnDFI is −0.126 significantly at the level of 0.01, which means that for every 1% increase in the DFI index, the urban–rural Theil index will decrease by 0.0013. Since the estimated coefficient of lnDFI in fixed effects model is only −0.057, the result means that DFI has a stronger effect on alleviating the urban–rural disparities in healthcare expenditures after adjusting for endogeneity.

#### 4.1.2 Robustness check

This section performed a series of tests to ensure robustness of the estimations. On the one hand, we replace the dependent variable with the ratio of healthcare expenditures between urban and rural residents. The result is shown in column (1) ~ (2) of Table 4. We can find that, similar to the estimated results of the baseline regression model, DFI also has a significant negative impact on the urban–rural ratio of healthcare expenditures. For the estimated coefficient, a 1% increase in the DFI index reduces the urban–rural ratio by 0.0060 and 0.0125 in the FE model and FE-IV model, respectively. On the other hand, we remove four special regions, i.e., Beijing, Tianjin, Chongqing, and Shanghai, which are municipalities directly under the Central Government. The impact of lnDFI on the Theil index of healthcare expenditures are re-estimated based on the adjusted sample still using both the FE model and FE-IV method. The results are presented in column (3) ~ (4) of Table 4, which present that lnDFI still has a significant negative effect on urban–rural disparities in healthcare expenditures and the value of the estimated coefficients has little difference from the baseline results. The above results indicate that our results are relatively robust using a different measurement of dependent variable and adjusting the research sample.

**Table 4:** Robustness tests.

	Replace the dependent variable		Remove the municipalities directly under the central government	
	Dependent variable: urban–rural ratio of per capita healthcare expenditures		Dependent variable: Theil index	
	(1) FE	(2) FE-IV	(3) FE	(4) FE-IV
lnDFI	−0.596*	−1.250*	−0.059**	−0.107*
	(0.317)	(0.719)	(0.025)	(0.056)
Control	Yes	Yes	Yes	Yes
FE of Province	Yes	Yes	Yes	Yes
FE of year	Yes	Yes	Yes	Yes
Wald-*F* statistics		334.085		287.721
Obs.	300	269	269	242
*R* ^2^	0.439	0.369	0.500	0.423

Clustering robust standard errors in brackets; *p < 0.1, **p < 0.05.

### 4.2 Effects of DFI sub-indices

Since the DFI index is a comprehensive index comprised of three sub-indices. Consequently, besides an estimate the impact of the overall DFI index on disparities in healthcare expenditures, we also explored the effects produced by the various sub-indices. We, respectively, used fixed effects models and Bartik instrumental variables for 2SLS estimation to explore the impact of the three indices.

Table 5 reports the regression results. The different dimensions of DFI have differentiated impacts on the urban–rural gap in healthcare expenditures. It can be found that the negative effect of DFI on urban–rural healthcare expenditure disparities mainly comes from its breadth of coverage, followed by its depth of usage. For the logarithmic breadth of coverage index, the estimated coefficient based on the fixed effects model is 0.019 (significant at the level of 0.01), and the coefficient increases to 0.064 (significant at the level of 0.05) after 2SLS estimation using instrumental variable. Although the estimated result of the depth of usage index based on the fixed effects model also reaches the significance level of 0.05, the coefficient is only significant at the level of 0.1 after endogeneity treatment. Different from the above two sub-indices, the degree of digitization is positively associated with urban–rural Theil index at the significance level of 0.05 in both models, which means that the digitization level of DFI widens the urban–rural gap in healthcare expenditures to some extent.

**Table 5:** Effects of DFI sub-indices on urban–rural disparities in healthcare expenditures.

	(1)	(2)	(3)	(4)	(5)	(6)
	FE	FE-IV	FE	FE-IV	FE	FE-IV
ln (breadth of coverage)	−0.019***	−0.064**				
	(0.007)	(0.027)				
ln (depth of usage)			−0.043**	−0.092*		
			(0.019)	(0.052)		
ln (degree of digitization)					0.025**	0.228**
					(0.011)	(0.110)
Control	Yes	Yes	Yes	Yes	Yes	Yes
FE of Province	Yes	Yes	Yes	Yes	Yes	Yes
FE of year	Yes	Yes	Yes	Yes	Yes	Yes
Wald-*F* statistics		636.983		75.463		19.512
Obs.	300	269	300	269	300	269
*R* ^2^	0.485	0.414	0.497	0.446	0.476	0.301

Clustering robust standard errors in brackets; *p < 0.1, **p < 0.05, ***p < 0.01.

### 4.3 Moderating effect of government health expenditures

To test whether the effect of DFI is related to government health expenditures, we performed a regression based on Equation (3). In the empirical operation, we decentralized the variables to avoid the multicollinearity problems which may be caused by the interaction terms. We further checked for multicollinearity effects by calculating the Variance Inflation Factors (VIF). The mean value of VIF is 3.53 (below the critical value of 5), which indicated no collinearities could be seen in the model.

The results are shown in Table 6. We can find that the mitigating effect of DFI on urban–rural healthcare expenditure disparities is consistent with that of the baseline model. Meanwhile, government health expenditures have a significant moderating effect at the significance level of 0.05. The negative coefficient of the interaction term indicates that, in the regions with more government health expenditures, DFI will have a stronger effect on alleviating the urban–rural disparities in healthcare expenditures.

**Table 6:** Moderating effect of government health expenditures on the impact of DFI on urban–rural disparities in healthcare expenditures.

	Theil index
lnDFI	−0.054*** (0.017)
lnDFI×lnHea	−0.025** (0.011)
lnHea	−0.084* (0.043)
Control	Yes
FE of Province	Yes
FE of year	Yes
Obs.	300
*R* ^2^	0.507

Clustering robust standard errors in brackets; *p < 0.1, **p < 0.05, ***p < 0.01.

## 5 Discussion

### 5.1 Main findings

Several salient findings were revealed. First, the DFI helps to bridge the urban–rural gap in healthcare expenditures, and this result is robust after taking the endogeneity concerns into account, changing the measurement of dependent variable and adjusting the research sample. Second, among the three sub-indices of DFI, only breadth of coverage has an obvious negative impact on the urban–rural gap in healthcare expenditures, while the depth of usage has a less significant negative impact and the degree of digitization even significantly widens the urban–rural gap in healthcare expenditures. Third, in the regions with more government health expenditures, the DFI has a stronger alleviating effect on the urban–rural disparities in healthcare expenditures.

The development of DFI plays an important role in narrowing the gap between urban and rural healthcare expenditures. Healthcare is a key input factor of health human capital. The elimination of the healthcare gap between urban and rural residents is beneficial for the equity of human capital, thus forming an internal motivation for the integrated development of urban and rural areas in the long run. The existing literature on DFI to alleviate urban–rural disparities mainly focuses on the aspects of income and total consumption. This finding provides new detailed empirical evidence for a comprehensive understanding of the role of DFI in bridging the gap between urban and rural areas in China from the perspective of healthcare expenditures.

We noted that the negative effect of DFI on urban–rural gap in healthcare expenditures mainly comes from its coverage breath instead of the other two sub-indices. For rural residents, most of them were excluded from the traditional financial system. The increase of breadth of coverage index means the growth in access to digital financial accounts. Rural residents lacking financial supports may experience a process to grow out of nothing in financial services after accessing digital accounts, which means an obvious marginal effect. For urban residents, DFI is more about transforming offline traditional financial mode into online form. Although the service convenience and universality have been improved, urban residents have already benefited from the development of traditional finance, so the influence of DFI is relatively weak. Therefore, the increase in breadth of coverage of digital finance can help narrow the gap between urban and rural healthcare expenditures. The increase of depth of usage index not only means more services are used, but also means the increase in service diversity. The increase in DFI service usage amount will obviously play a certain role in alleviating the urban–rural healthcare gap. However, considering that compared to urban residents, rural residents are relatively lack of financial knowledge due to their limited education and economic situation, the improvement of service diversity may not have as obvious benefits for rural residents. Furthermore, it should be noted that the digitization level of DFI has exacerbated the inequality in healthcare expenditures between urban and rural areas on the contrary. Zhou and Chen (37) also drew a similar conclusion from the study on the impact of DFI on the urban–rural disparities of total consumption expenditure. The digital transformation of Chinese financial system started relatively late, the digital financial resources are limited and the allocation is not balanced (38). Rural areas face the problem of insufficient digital infrastructure construction, and the digital tools popularity and digital literacy of rural residents are significantly weaker than those of urban residents. As a result, the improvement effect of digitization level on urban healthcare expenditures is greater than that of rural residents. Existing studies have also shown that although DFI helps rural development, it puts forward higher requirements for digital infrastructure construction. Once these requirements are not met, the “digital divide” will become prominent (39), thus producing the so-called “Matthew effect” (i.e., the stronger becomes stronger and the weaker becomes weaker), which greatly limits the benefits of rural households in the development of DFI. Our results show that DFI still has deficiencies in the inclusion of rural residents in the aspect of digital development, which cannot be ignored in the process of promoting urban and rural integration.

We further explored some more details of the impact of DFI on the urban–rural gap in healthcare expenditures, and found that the increase of government health expenditures contributes to the DFI’s effect. The increase of government health expenditures, especially on the basic medical security system and the medical service supply system, greatly enhances the access of rural residents to healthcare products and services. The results of baseline model mainly verify that DFI can reduce the urban–rural disparities in healthcare expenditures from the demand-side; while the moderating effect model shows that government health expenditures further strengthen the beneficial effect of DFI from the supply-side by improving the accessibility of rural residents to affordable quality medical resources. This finding provides a more detailed basis for promoting the equalization of healthcare in urban and rural areas in the digital era. It reminds policy makers that more attention should be paid to the role of government as a key role in future reforms. Considering the current situation of imbalanced allocation of medical resources between urban and rural areas in China, (data from China Statistics Yearbook presents that till the year of 2022 the number of health professionals and beds in medical institutions per thousand population in rural areas was 10.20 and 7.66, respectively, while in rural areas it was only 6.55 and 6.25), the government need to increase financial support for medical resources in rural areas. Then it can be expected that urban–rural gap in healthcare utilization will be further narrowed in the future with the effect of DFI.

### 5.2 Limitations

There were several potential limitations to this paper. First, the independent variable is the DFI Index compiled by other researchers, which means it is a second-hand database. Although this index has been widely used in related research, it still inevitably leads to the possibility that our research results may be affected by its reliability. Moreover, the index is only based on the micro-data from Ant Financial Services Group. Although Ant Group is a dominant firm in China’s digital financial industry, with the development of DFI, some similar firms also emerged and the traditional financial institutions have gradually started similar businesses. This may result in the index not being able to fully reflect the development status of DFI in China. Second, due to data limitations, we are unable to fully control the disparities in healthcare needs between urban and rural residents. But in order to minimize the impact of healthcare needs, we have controlled the population’s age structure and air pollution at the overall level of provinces. Third, this study used provincial-level data, so the individual-level mechanisms were not explored. Since DFI includes a variety of digital financial services, we will conduct more detailed research based on the usage of specific services by individuals in the future.

## 6 Conclusion

The study reveals that DFI plays an important role in bridging the urban–rural gap in healthcare expenditures. Policymakers should continue to promote the development of DFI in China. While the degree of digitization is the major constraint on the urban–rural inclusion effect of DFI. To solve this problem, the government needs to accelerate the construction of digital infrastructure and increase the penetration rate of digital tools in rural areas.

We also find that the increase in government health expenditures promotes DFI to reduce the urban–rural disparities in healthcare expenditures. This is suggestive evidence for the synergistic role of government in promoting the beneficial impact of DFI. It highlights the importance of demand–supply matching. According to data from the China Statistical Yearbook, the urban–rural imbalance in the allocation of medical and health technical personnel is particularly prominent. The local government should address the shortage of rural human resources in healthcare through diverse incentive policies. As DFI drives rural residents’ healthcare demand, the unbalanced allocation of medical resources between urban and rural areas should be addressed at a faster pace.

This study also provides a few insights for future research. Firstly, since DFI can effectively alleviate the disparities of access to healthcare services, it may also have an impact on health inequality or disease prevention. People with lower economic status are more susceptible to many diseases (e.g., tuberculosis, malaria…) and have relatively limited access to medical resources. The economic improvements brought about by DFI may reduce the incidence of diseases among the poor, and less inequality in healthcare utilization is also expected to improve their cure rate. For some highly communicable epidemics such as COVID-19, effectively reducing the infections of vulnerable groups may even contribute to preventing these diseases within the whole society. In addition, this paper and a series of similar studies on DFI primarily focus on its short-term impact on welfare, while its potential long-term economic consequences have not been fully discussed. It is worth noting that, while inclusive strategies focusing on credit and government expenditure can improve the welfare of certain groups in the short term, it usually means that another group have to bear the corresponding costs in the long term, such as through invisible taxes and indirect appropriation. So we consider that future research could pay more attention to the long-term economic and fiscal consequences of such public policies, and what measures policymakers should take to address these consequences.

## Data availability statement

Publicly available datasets were analyzed in this study. This data can be found here: https://idf.pku.edu.cn.

## Author contributions

YZ: Conceptualization, Data curation, Funding acquisition, Formal analysis, Writing – original draft, Writing – review & editing. KL: Data curation, Writing – original draft. YP: Methodology, Supervision, Funding acquisition, Writing – review & editing. PC: Conceptualization, Writing – review & editing.

## Footnotes

^1^http://iif.pku.edu.cn

## References

[1] Allison RA Foster JE. Measuring health inequality using qualitative data. J Health Econ. (2004) 23:505–24. doi: 10.1016/j.jhealeco.2003.10.006 15120468

[2] Baeten S Van Ourti T Van Doorslaer E. Rising inequalities in income and health in China: who is left behind? J Health Econ. (2013) 32:1214–29. doi: 10.1016/j.jhealeco.2013.10.002, PMID: 24189450 PMC3880577

[3] Li Y Sun Y Zhang Y Yi D Ma C Ma S. Rural–urban disparity in health care: observations from Suzhou China. Public Health. (2016) 138:164–7. doi: 10.1016/j.puhe.2016.03.026 27137871

[4] Harris B Goudge J Ataguba JE McIntyre D Nxumalo N Jikwana S. Inequities in access to health care in South Africa. J Public Health Policy. (2011) 32:S102–23. doi: 10.1057/jphp.2011.35 21730985

[5] Zhang C Zhu Y Zhang L. Effect of digital inclusive finance on common prosperity and the underlying mechanisms. Int Rev Financ Anal. (2024) 91:102940. doi: 10.1016/j.irfa.2023.102940

[6] Clarke G Xu L Zou H. Finance and income inequality: test of alternative theories. An Econ Financ. (2013) 14:493–510. doi: 10.2139/ssrn.364160

[7] Neaime S Gaysset I. Financial inclusion and stability in MENA: evidence from poverty and inequality. Financ Res Lett. (2018) 24:230–7. doi: 10.1016/j.frl.2017.09.007

[8] Jeanneney SG Kpodar K. Financial development and poverty reduction: can there be a benefit without a cost? J Dev Stud. (2011) 47:143–63. doi: 10.1080/00220388.2010.506918

[9] Zhang X Wan G Zhang J He Z. Digital economy, inclusive finance, and inclusive growth. Econ Res. (2019) 54:71–86.

[10] Zhang X Zhang J Wan G Luo Z. Fintech growth and inequality: evidence from China’s household survey data. Singap Econ Rev. (2020) 65:75–93. doi: 10.1142/S0217590819440028

[11] Luo J Li B. Impact of digital financial inclusion on consumption inequality in China. Soc Indic Res. (2022) 163:529–53. doi: 10.1007/s11205-022-02909-6

[12] Meyer BD Sullivan JX. Measuring the well-being of the poor using income and consumption. J Hum Resour. (2003) 38:1180–220. doi: 10.2307/3558985

[13] Deaton A Paxson C. Intertemporal choice and inequality. J Polit Econ. (1994) 102:437–67. doi: 10.1086/261941

[14] Li J Yu W Jing J. The impact of digital finance on household consumption: evidence from China. Econ Model. (2020) 86:317–26. doi: 10.1016/j.econmod.2019.09.027

[15] Zhang Y Hu W Li J. Research on the impact of digital inclusive finance on the consumption gap between urban and rural residents. Macroecon Res. (2022) 44:51–63. doi: 10.16304/j.cnki.11-3952/f.2022.04.004

[16] Zhang H Han X. Can digital finance alleviate major social contradictions? The perspective of consumption inequality. Econ Sci. (2022) 44:96–109.

[17] Levchenko A. Financial liberalization and consumption volatility in developing countries. IMF Econ Rev. (2005) 52:237–59. doi: 10.2307/30035897

[18] He Z Song X. How does the development of digital finance affect residents’ consumption. Financ Trade Econ. (2020) 41:65–79. doi: 10.19795/j.cnki.cn11-1166/f.2020.08.005

[19] Yi X Zhou L. Whether the development of digital inclusive finance significantly affects household consumption: Micro evidence from Chinese households. Financ Res. (2018) 61:47–67.

[20] Zhang T Cai K. Has digital inclusive finance narrowed the consumption gap between urban and rural residents? Empirical test based on China’s provincial panel data. Econ Problems. (2021) 43:31–9. doi: 10.16011/j.cnki.jjwt.2021.09.004

[21] Li Z Zheng J. The impact of digital inclusive finance on the consumption gap between urban and rural residents. Mod Econ Res. (2023) 42:42–50. doi: 10.13891/j.cnki.mer.2023.06.009

[22] Grossman M. On the concept of health capital and the demand for health. J Polit Econ. (1972) 80:223–55. doi: 10.1086/259880

[23] Rosett RN Huang L. The effect of health insurance on the demand for medical care. J Polit Econ. (1973) 81:281–305. doi: 10.1086/260028

[24] Fleurbaey M Schokkaert E. Unfair inequalities in health and healthcare. J Health Econ. (2009) 28:73–90. doi: 10.1016/j.jhealeco.2008.07.016 18829124

[25] Pierrakis Y Collins L. Crowdfunding: a new innovative model of providing funding to projects and businesses. SSRN [preprint]. (2013). Available at: https://ssrn.com/abstract=2395226 (Accessed May 05, 2013).

[26] Yu C Jia N Li W Wu R. Digital inclusive finance and rural consumption structure – evidence from Peking University digital inclusive financial index and China household finance survey. China Agric Econ Rev. (2022) 14:165–83. doi: 10.1108/CAER-10-2020-0255

[27] Xu Y Peng Z Sun Z Zhan H Li S. Does digital finance lessen credit rationing? – evidence from Chinese farmers. Res Int Bus and Financ. (2022) 62:101712. doi: 10.1016/j.ribaf.2022.101712

[28] Ahmed H Cowan BW. Mobile money and healthcare use: evidence from East Africa. World Dev. (2021) 141:105392. doi: 10.1016/j.worlddev.2021.105392

[29] Lv G Liu W. Mobile payment, medical infrastructure and rural residents’ redical services utilization. Soc Sci. (2022) 4:95–105. doi: 10.13262/j.bjsshkxy.bjshkx.220411

[30] Han Y Li C. Does mobile payment promote family medical and health expenditure? Financ Econ. (2019) 9:50–6. doi: 10.19622/j.cnki.cn36-1005/f.2019.09.008

[31] Andersen RM. Revisiting the behavioral model and access to medical care: does it matter? J Health Soc Behav. (1995) 36:1–10. doi: 10.2307/2137284 7738325

[32] Guo F Wang J Wang F Kong T Zhang X Cheng Z. Measuring the development of digital inclusive finance in China: index compilation and spatial characteristics. China Econ Q. (2020) 19:1401–18. doi: 10.13821/j.cnki.ceq.2020.03.12

[33] Ji X Wang K Xu H Li M. Has digital financial inclusion narrowed the urban-rural income gap: the role of entrepreneurship in China. Sustain For. (2021) 13:8292. doi: 10.3390/su13158292

[34] Theil H. Economics and information theory, North Holland Publishing Company: Amsterdam Netherlands (1967), 132–139.

[35] Gao F. The inflection point of China’s urban and rural consumption and its economic growth effect. Stat Res. (2014) 31:41–6. doi: 10.19343/j.cnki.11-1302/c.2014.12.006

[36] Bartik TJ. How do the effects of local growth on employment rates vary with initial labor market conditions? Policy Paper No. 2009–005. Kalamazoo, MI: W.E. Upjohn Institute for Employment Research (2006).

[37] Zhou L Chen Y. Digital inclusive finance and urban-rural income gap: theoretical mechanism empirical evidence and policy choice. World Econ Res. (2022) 38:117–34. doi: 10.13516/j.cnki.wes.2022.05.004

[38] Wang Y Tan Z Zheng L. Research on the impact of digital inclusive finance on social security. J Quant and Technol Econ. (2020) 37:92–112. doi: 10.13653/j.cnki.jqte.2020.07.005

[39] Xing Y. The “dividend” and “gap” of rural digital inclusion finance. Economist. (2021) 33:102–11. doi: 10.16158/j.cnki.51-1312/f.2021.02.011

